# Microsecond protein dynamics observed at the single-molecule level

**DOI:** 10.1038/ncomms8685

**Published:** 2015-07-07

**Authors:** Takuhiro Otosu, Kunihiko Ishii, Tahei Tahara

**Affiliations:** 1 Molecular Spectroscopy Laboratory, RIKEN, 2-1, Hirosawa, Wako 351-0198, Japan; 2 Ultrafast Spectroscopy Research Team, RIKEN Center for Advanced Photonics (RAP), RIKEN, 2-1, Hirosawa, Wako 351-0198, Japan

## Abstract

How polypeptide chains acquire specific conformations to realize unique biological functions is a central problem of protein science. Single-molecule spectroscopy, combined with fluorescence resonance energy transfer, is utilized to study the conformational heterogeneity and the state-to-state transition dynamics of proteins on the submillisecond to second timescales. However, observation of the dynamics on the microsecond timescale is still very challenging. This timescale is important because the elementary processes of protein dynamics take place and direct comparison between experiment and simulation is possible. Here we report a new single-molecule technique to reveal the microsecond structural dynamics of proteins through correlation of the fluorescence lifetime. This method, two-dimensional fluorescence lifetime correlation spectroscopy, is applied to clarify the conformational dynamics of cytochrome *c*. Three conformational ensembles and the microsecond transitions in each ensemble are indicated from the correlation signal, demonstrating the importance of quantifying microsecond dynamics of proteins on the folding free energy landscape.

## Abstract (web summary)

Single molecule spectroscopy can visualise dynamic changes in protein conformation on the submillisecond timescale. Here, Otosu *et al*. apply two-dimensional fluorescence lifetime correlation spectroscopy to visualise dynamics between seven conformers of cytochrome *c* on the microsecond timescale.

## Graphical abstract

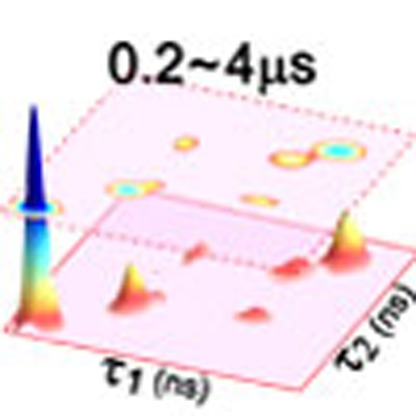

Conformational heterogeneity and structural fluctuation are essential molecular properties of proteins^1^,^2^. Because they are not usually detectable in ordinary ensemble-averaged measurements, the single-molecule fluorescence resonance energy transfer (smFRET) technique is an indispensable tool for studying the molecular-level properties of proteins^3^. In particular, smFRET experiments with high time resolution are of emerging importance because the experimental data can be directly compared with all-atom molecular dynamics simulations for timescales up to the millisecond range^4^,^5^. The qualitative comparison between experiments and computations provides atomic details of structural dynamics of proteins^5^,^6^,^7^, which facilitates accurate understanding of how proteins realize their functions.

Protein folding is a central problem for which conformational heterogeneity and structural fluctuation are extensively studied. Particularly, the microsecond dynamics of protein folding have attracted much interest recently. In fact, the ‘speed limit' of protein folding is supposed to be ∼1 μs and it closely relates to the roughness of the folding free energy landscape as well as to the internal friction of the polypeptide chain in the unfolded state^8^,^9^,^10^,^11^. Furthermore, microsecond structural fluctuations in the native and intermediate states are highly relevant to protein functions, as well as protein–protein interactions^12^,^13^. Therefore, elucidation of the microsecond conformational dynamics of proteins is critically important for obtaining a comprehensive understanding of the folding processes of proteins.

In spite of the importance of microsecond dynamics of proteins, application of the conventional smFRET technique is limited to dynamics slower than ∼100 μs because of the difficulty in collecting a sufficient number of photons to evaluate the FRET efficiency in a short bin time^14^. This time resolution is insufficient to quantitatively examine the complete conformational dynamics of proteins. Several attempts have been made recently to detect submicro to microsecond dynamics using smFRET techniques^15^, and the transition path time between substates^16^ and the reconfiguration time within a long-lived substate^17^ have been examined. However, it is still very challenging to detect transient states with a microsecond time resolution and to quantify the hierarchical energy landscape of the protein folding.

Here, we report on a new single-molecule spectroscopic method, two-dimensional fluorescence lifetime correlation spectroscopy (2D FLCS), to quantitatively elucidate the microsecond conformational dynamics of proteins^18^,^19^,^20^. This method is an extension of fluorescence correlation spectroscopy, in which the correlation of the fluorescence lifetime is detected. Because the fluorescence lifetime can be used as a molecular ruler in FRET^21^, different conformations (conformers) are distinguished through their distinct fluorescence lifetimes at the single-molecule level. We apply 2D FLCS to elucidate the spontaneous conformational dynamics of yeast iso-1-cytochrome *c* (cyt *c*) in acidic conditions. The fluorescence lifetime correlation reveals the highly heterogeneous nature of cyt *c* and clarifies a variety of conformational dynamics including the transitions between conformers on a timescale of several microseconds.

## Results

### 2D fluorescence lifetime correlation spectroscopy

2D FLCS utilizes the fluorescence lifetime of a FRET donor as a ruler for measuring the protein structure. The FRET efficiency between a pair of dye molecules is represented using the donor fluorescence lifetime as follows:


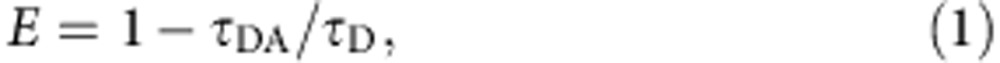


where *τ*_DA_ and *τ*_D_ represent the donor lifetimes in the presence and absence of the acceptor, respectively. The donor fluorescence lifetime is measured using the time-correlated single photon-counting (TCSPC) method, in which the emission delay time (*t*) of each signal photon with respect to the corresponding excitation pulse is recorded and histogrammed. From the obtained emission delay histogram, *I*(*t*), the fluorescence lifetime distribution, *α*(*τ*), is derived using the following Laplace transform relationship.


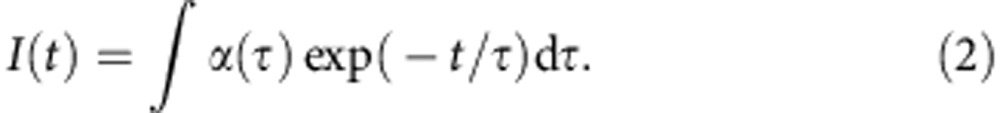


The fluorescence lifetime is determined as a peak position of the *α*(*τ*).

**Figure 1:**
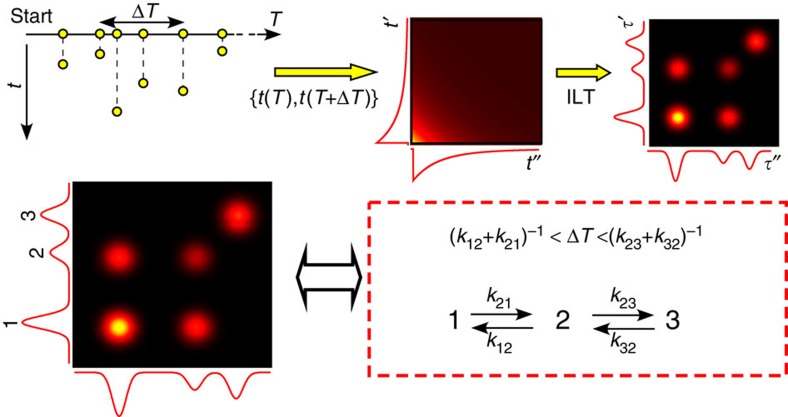
Concept of two-dimensional fluorescence lifetime correlation spectroscopy. Schematic illustration of two-dimensional fluorescence lifetime correlation spectroscopy. *T* is the absolute arrival time of a photon from the start of an experiment, *t* is the emission delay time with respect to the corresponding excitation pulse, and ILT stands for inverse Laplace transform.

In 2D FLCS, a pair of emission delay times (*t*′*, t″*) of photon pairs, which are temporally separated by a certain interval (Δ*T*), is accumulated to construct a 2D emission-delay correlation map (Fig. 1). With subtraction of the uncorrelated background, the obtained 2D map represents the correlation of fluorescence decay curves measured with a time interval of Δ*T*. A 2D inverse Laplace transform (ILT) is then applied to this 2D map to convert the coordinates from the emission delay time to the fluorescence lifetime. In the obtained 2D lifetime correlation map, the diagonal peaks show how many fluorescence lifetime components (that is, how many conformers showing distinct FRET efficiencies) are contained in the correlation function, while off-diagonal peaks indicate how these lifetime components (that is, conformers) are correlated to each other within Δ*T.*

### pH-induced conformational diversity of cyt *c*

Cyt *c* is a small haem protein which functions as an electron transporter^22^. The folding mechanism of this protein has been extensively studied at the ensemble-average level, as well as at the single-molecule level^23^,^24^,^25^,^26^. In the present work, one fluorophore, Alexa546, is attached as a donor to the single free cysteine residue of cyt *c* (C102) that is located in the C-terminal region. The fluorescence spectrum of Alexa546 has a substantial overlap with the visible absorption band of haem in cyt *c*. Therefore, the conformational heterogeneity and their transition dynamics of cyt *c* are evaluated through the change in the FRET efficiency between Alexa546 and the haem.

**Figure 2:**
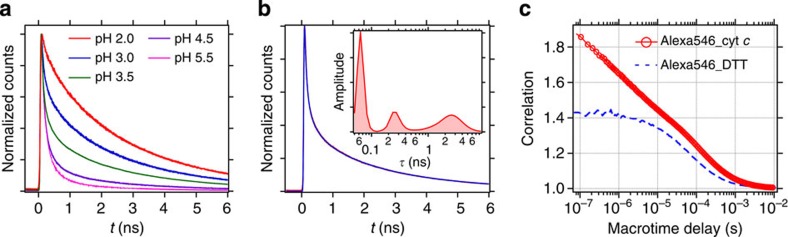
Fluorescence decay and fluorescence correlation curves. (**a**) pH-dependent fluorescence decay curves of Alexa546_cytochrome *c*. (**b**) Fluorescence decay curve of Alexa546_cytochrome *c* at pH 3.5 (red solid line). Inverse Laplace transform was performed and the result is shown in the inset. The decay curve calculated by Laplace transform from the lifetime distribution is also shown (blue solid line). Decay curves in **a**,**b** are normalized for the intensity at *t*=0. (**c**) Fluorescence correlation curve (red circle) of Alexa546_cytochrome *c* at pH 3.5. The data obtained from Alexa546_DTT at the same pH is also shown for comparison (blue broken line).

Figure 2a shows the pH-dependent change in the fluorescence decay of Alexa546 in cyt *c* (Alexa546_cyt *c*). In the neutral pH condition, the fluorescence of Alexa546_cyt *c* decays rapidly. The fluorescence lifetime becomes longer as pH is shifted to the acidic side. Because cyt *c* undergoes pH-induced unfolding^24^,^27^, the change in the donor fluorescence lifetime reflects the pH-induced conformational change of cyt *c* that changes the distance and FRET efficiency between Alexa546 and haem. For the data taken at pH 3.5, ILT was performed to obtain the lifetime distribution in the fluorescence decay (Fig. 2b). The obtained lifetime distribution (inset in Fig. 2b) shows several distinct peaks, suggesting that there are several fluorescence lifetime components in the fluorescence decay kinetics of Alexa546_cyt *c* at pH 3.5. In other words, cyt *c* takes several conformations at this pH, each of which has a unique fluorescence lifetime (that is, a donor–haem distance).

The transition dynamics between cyt *c* conformers at pH 3.5 was first examined by conventional FCS, and the obtained correlation curve is shown in Fig. 2c. The data of Alexa546 reacted with dithiothreitol (DTT) (Alexa546_DTT) are also shown as a reference. The correlation curve of Alexa546_cyt *c* shows a structureless decay feature over 3 orders of timescale (100 ns–10 μs) that cannot be described with a single-exponential function. This suggests that the complex kinetics due to protein dynamics is observed in the correlation function on this timescale. Lifetime-weighted FCS analysis^28^ of Alexa546_cyt *c* at pH 3.5 was also performed. The results indicate that the transition dynamics occur on a ∼5 μs timescale (Supplementary Fig. 1).

### 2D map reveals dynamics among five conformers

**Figure 3:**
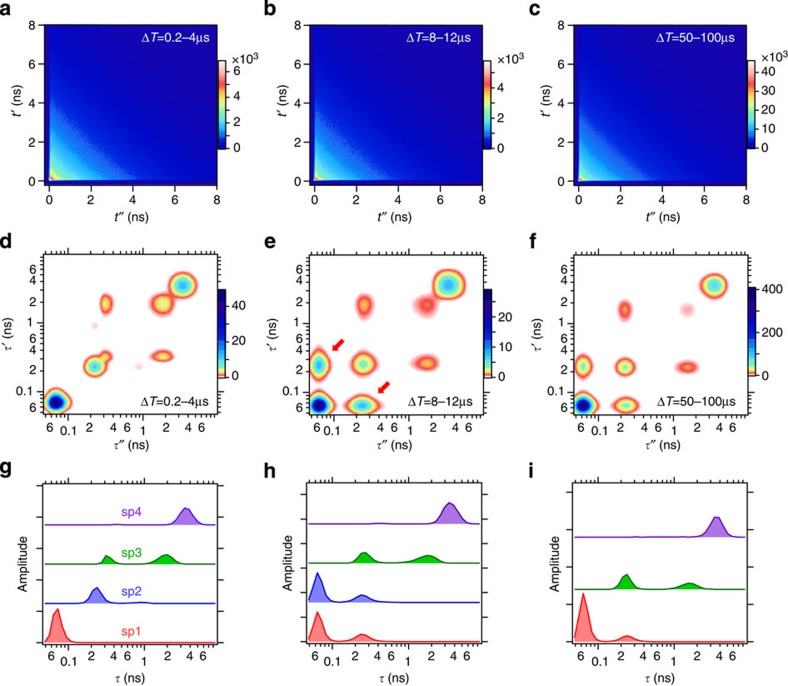
Two-dimensional fluorescence lifetime correlation spectroscopy of Alexa546_cytochrome *c*. 2D emission-delay correlation maps (**a**–**c**), 2D lifetime correlation maps (**d**–**f**) and independent fluorescence lifetime distributions (**g**–**i**), which are calculated for Δ*T*=0.2–4 μs (**a**,**d**,**g**), 8–12 μs (**b**,**e**,**h**) and 50–100 μs (**c**,**f**,**i**). *t*′ and *τ*′ are the microtime and the fluorescence lifetime of the first photons, and *t*″ and *τ*″ are those of the second photons in photon pairs, respectively. The arrows in **e** indicate the cross peaks. The 2D lifetime correlation maps are smoothed using a spline for visual clarity. In the measurements, excitation is at 540 nm and pH is 3.5.

Figure 3 shows representative 2D emission-delay correlation maps and 2D lifetime correlation maps evaluated for three different Δ*T*. The 2D lifetime correlation maps are described as a sum of the contributions from several fluorescence lifetime distributions. Four independent lifetime distributions (denoted sp1, sp2, sp3 and sp4 in Fig. 3g) are required to reproduce the 2D emission-delay correlation map at the shortest Δ*T*, 0.2–4 μs. Assignments of the observed fluorescence lifetime components were made based on global fitting analysis of fluorescence decay curves obtained with different solvent conditions (Supplementary Fig. 2, and Supplementary Note 1). From the analysis, we assign the shortest lifetime component (70 ps in sp1) to the native state (N), and the longest lifetime component (3.3 ns in sp4) to the unfolded state (U). The other three lifetime components (280 ps in sp2, 300 ps in sp3 and 1.7 ns in sp3) are attributed to the folding intermediate states of cyt *c* (I_1_, I_2_ and I_3_, respectively). The lifetime distribution of sp3 contains two lifetime components. They represent the lifetimes of I_2_ and I_3_, and the corresponding cross peaks appear even in the 2D lifetime correlation map calculated at Δ*T*=0.2–4 μs (Fig. 3d). This indicates that the interconversion between these two lifetime components occurs faster than this Δ*T.* In other words, the conformational transition between I_2_ and I_3_ takes place faster than ∼1 μs.

In the 2D lifetime correlation map at Δ*T*=8–12 μs (Fig. 3e), the cross peaks are observed between 70 ps (N) and 280 ps (I_1_) components as indicated by arrows. These cross peaks are missing in the map at Δ*T*=0.2–4 μs (Fig. 3d, Supplementary Fig. 3 and Supplementary Note 2). This is clear evidence that sp1 (or N) and sp2 (or I_1_) are equilibrated on this timescale. In other words, the conformational transition between N and I_1_ occurs in several microseconds. Thus, the sp1 and sp2 become indistinguishable on this timescale and the number of the independent lifetime distributions is reduced to 3 (Supplementary Note 3).

**Figure 4:**
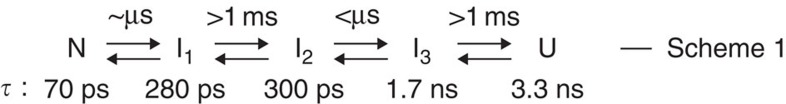
Folding scheme of cytochrome *c*. The timescales for the conformational transition and the fluorescence lifetimes of each substate are also shown.

No further significant change was observed in the 2D lifetime correlation maps from 50 μs up to 1 ms (Fig. 3f and Supplementary Fig. 4). This indicates that other equilibration processes, for example, equilibration between N and U, occur on a timescale longer than 1 ms. Taken together, the conformational transition scheme of cyt *c* is obtained, assuming that the intermediate states found in the present study are on the folding pathway (Fig. 4). It may be worth noting that, however, the schemes involving off-pathway or parallel pathway cannot be excluded at the moment because the misligation states may appear as off-pathway intermediates in cyt *c*^29^.

### Detection of hidden conformers

In a 2D lifetime correlation map, the diagonal peaks and the off-diagonal peaks (cross peaks) represent autocorrelations and cross-correlations of corresponding lifetime components, and the peak intensity is proportional to the correlation value at selected Δ*T*. Therefore, the correlation functions of each lifetime component can be extracted from a series of 2D lifetime correlation maps at different Δ*T* values (Supplementary Note 4). The autocorrelations of four substates (N, I_1_, I_en_=I_2_+I_3_, and U) and the cross-correlation between N and I_1_ are obtained from 2D lifetime correlation maps at various Δ*T* up to Δ*T*=0.7–1.0 ms (Supplementary Figs 4 and 5). Note that I_2_ and I_3_ are analysed as I_en_ for further analysis because they are already equilibrated at the shortest Δ*T*.

**Figure 5:**
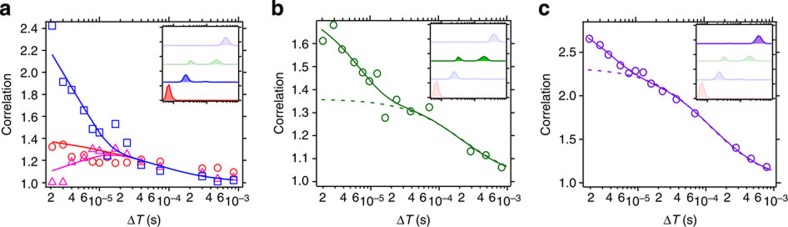
Correlation curves of substates of cytochrome *c*. (**a**) Autocorrelations of N (red circles), I_1_ (blue squares), and the cross-correlation between N and I_1_ (pink triangles). (**b**) Autocorrelation of I_en_ (green circles). (**c**) Autocorrelation of U (purple circles). The corresponding lifetime component is highlighted in the inset of each figure. The correlation curves in **a** were fitted with theoretical equations (20)–(22), 33, 34, and the best fits are shown with solid lines. For the data shown in **b**,**c**, each data set was fitted both with equations (25) and (26), and the fits are shown with broken and solid lines, respectively.

The autocorrelations and the cross-correlation of N and I_1_ show different values in the microsecond Δ*T* region, but they gradually converge to an identical curve at longer Δ*T* (Fig. 5a). These data are fitted with the theoretical equations that take account of the conformational transition between N and I_1_. The correlation curves are well fitted with equations derived from the two-state model (Fig. 5a), confirming the validity of Scheme 1 shown in Fig. 4. The equilibration time between N and I_1_ is determined to be 5 μs from this fitting.

The autocorrelations of I_en_ and U are also calculated (Fig. 5b,c). From Scheme 1, it is anticipated that the autocorrelations of these lifetime components can be described with a simple model based on translational diffusion in and out of the focus region. However, this model does not reproduce the autocorrelation functions of these lifetime components (broken lines in Fig. 5b,c). In contrast, the correlation curves are well fitted with the equation that includes a conformational equilibration term (solid lines in Fig. 5b,c). Because the 2D lifetime correlation map does not show any signatures indicating the equilibration process of I_en_ and U with other substates, this indicates that highly quenched ‘dark' states (denoted as D) are involved in the conformational dynamics of this protein. In fact, if the fluorescence lifetimes of dark states are much shorter than the time resolution of the measurement, they will not appear in the 2D lifetime correlation map, that is, no corresponding peaks appear. However, conformational transition dynamics between an emissive state and a dark state induce blinking of the fluorescence intensity without changing the fluorescence lifetime of the emissive states. Thus, it affects the fluorescence correlation curve as an additional decay component. We confirm the involvement of the dark states by analysing the pH effect on the fluorescence correlation curves and fluorescence decay curves (Supplementary Fig. 6 and Supplementary Note 5). In such a dark state, cyt *c* presumably takes a conformation in which the specific interaction between Alexa546 and aromatic amino acid residues becomes predominant, resulting in very fast quenching of the fluorescence^30^,^31^. The equilibration time between I_en_ and D_1_ as well as that between U and D_2_ were determined from the fitting of the correlation curves of I_en_ and U, respectively.

**Figure 6:**
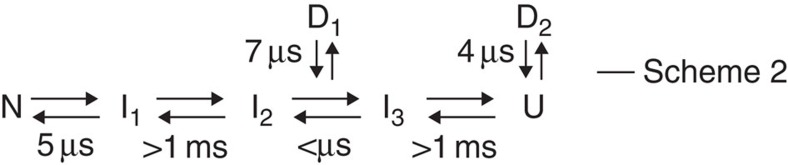
Full folding scheme of cytochrome *c*. The timescales for the conformational transition are also shown.

Based on these analyses of the correlation curve of each lifetime component identified by 2D FLCS, we can refine the conformational transition scheme of cyt *c*, by including two dark states (Fig. 6). The validity of the proposed model is confirmed by the fitting of the total correlation function (Fig. 2c) using theoretical formula based on Scheme 2 (Supplementary Fig. 7).

## Discussion

**Figure 7:**
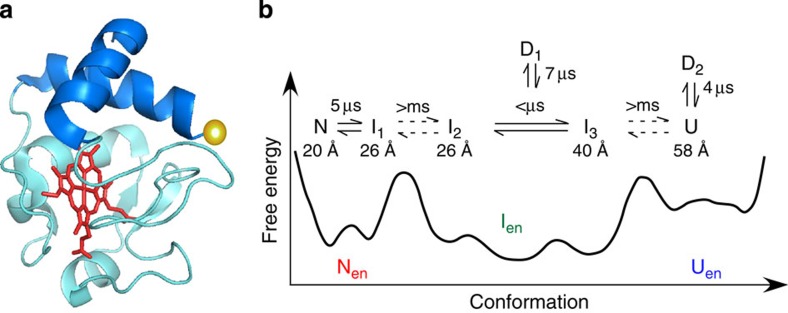
Conformational transition of cytochrome *c.* (**a**) Native-state structure of cytochrome *c* (PDB ID:1YCC). N- and C-terminal helices are highlighted with blue. The position of the donor dye is shown by a yellow sphere. (**b**) Schematic free energy landscape and relevant conformational dynamics of cytochrome *c* (pH 3.5). The equilibration times among conformers and the donor–haem distances evaluated in the present work are given.

The present study at an acidic condition has identified seven different conformers on the folding energy landscape of cyt *c* (Fig. 6). Considering the transition dynamics observed, they can be divided into three groups, that is, N ensemble (N_en_: N and I_1_), I ensemble (I_en_: I_2_, I_3_ and D_1_) and U ensemble (U_en_: U and D_2_). 2D FLCS clearly shows that the conformational transitions within each ensemble occur on the microsecond or shorter timescale, whereas those between different ensembles take longer than a millisecond. This implies that each of these ensembles is separated by a substantial potential barrier on the energy landscape and is kinetically trapped in a local region with a moderate depth (Fig. 7b). This three-state model is in good agreement with previous ensemble-averaged studies^32^. A single-molecule study using a similar donor dye attached to the same position also indicated the existence of a folding intermediate^33^.

Assuming that FRET from Alexa546 to the haem solely determines the fluorescence lifetime, the fluorescence lifetime of each conformer can be a measure of the donor–haem (D–A) distance (*r*) through Förster equation,


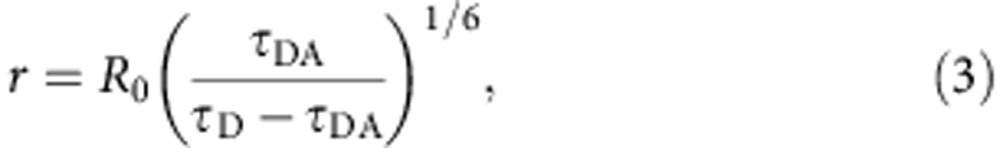


where *R*_0_ is the Förster distance (41 Å in this FRET pair), and *τ*_DA_ and *τ*_D_ (=4.0 ns) are the donor fluorescence lifetimes in the presence and absence of the acceptor, respectively. The D–A distances calculated for each conformer allow us to discuss the conformational transition of cyt *c* in more detail.

The large fluorescence lifetime difference between U and I_3_ indicates that the initial rate-limiting process of the folding (which is followed by the fast conformational change within I_en_, that is, I_3_ to I_2_) includes a large-scale contraction of the donor–haem distance. Because the donor dye is attached on C terminus, this large change in the donor–haem distance is attributable to a large conformational change in the C-terminal domain, such as α-helix formation. A previous thorough hydrogen-exchange study showed that the N- and C-terminal helices (blue in Fig. 7a) are energetically the most stable, and they are expected to fold in the earliest stage of the process^34^. A submillisecond-time-resolved CD measurement showed that substantial amounts of helical structure are formed after the initial rate-limiting step, and it was argued that specific interaction between the N- and C-terminal domains induces the formation of helices in these regions^35^. Furthermore, it was demonstrated that when the N- and C-terminal segments are chemically connected with S–S bonding, the α-helix content in these regions is increased^36^. These studies suggest that the transition between U and I_3_, and that between I_3_ and I_2_ are highly likely attributable to contact between the N- and C-terminal domains (U→I_3_), and the subsequent α-helix formation or stabilization (I_3_→I_2_). Submicrosecond dynamics between I_3_ and I_2_ does not contradict the previous observations that α-helix formation of synthetic peptides takes place in the submicrosecond to microsecond timescale^9^.

The fluorescence lifetimes of I_1_ and I_2_ are almost the same, and the conformational transition between I_1_ and I_2_ does not show any detectable change in the D–A distance. The evaluated D–A distances of I_1_ and I_2_ (26 Å) are close to that of N (20 Å), indicating that the local configuration around the haem moiety and C-terminal helix is preserved during I_1_ and I_2_. Therefore, the transition from I_2_ to I_1_ is likely due to the folding of other domains that are distant from the N- and C-terminal domains. Although the origin of the large energetic barrier between I_1_ and I_2_ is not clear at the moment, combination of 2D FLCS and protein engineering that inserts the donor at different positions will reveal the conformational origin of this rate-limiting step in the folding of cyt *c*.

2D FLCS clearly shows the microsecond conformational transition in N_en_ by the appearance of cross peaks between N and I_1_. This conformational transition is of particular interest because it corresponds to the final stage of the folding process. The moderate change (∼6 Å) in the D–A distance suggests that this transition highly likely arises from the folding of distant regions. We note that a native-state hydrogen-exchange study suggested that the final folding process of cyt *c* is the formation of several Ω loops that are distant from the C-terminal helix^37^. The time constant (5 μs) of the transition between N and I_1_ looks consistent with this assignment because, although the end-to-end contact formation of smaller synthetic peptides often occurs on a submicrosecond timescale^9^, it is known that the loop formation in proteins takes much longer^38^. In fact, very recently, an all-atom molecular dynamics simulation indicated that the loop formation in the native-state ensemble of ubiquitin occurs on a microsecond timescale^5^. The conformational heterogeneity and the dynamics in the native state are directly related to how proteins utilize their structural fluctuations to realize their functions, such as recognition and binding of targets^39^. Therefore, finding this microsecond conformational dynamics in N_en_ is very important for elucidating the functional mechanism of cyt *c*.

So far, kinetic studies of protein folding on the nanosecond–microsecond timescales have been mainly performed by laser-induced temperature-jump techniques^40^. However, in these perturbation-induced kinetic measurements, fast dynamics among different conformers that occurs after a slower rate-limiting step cannot be detected. Single-molecule measurements overcome this problem, but conventional techniques do not provide sufficient time resolution and/or high quality data for rigorous quantitative analysis. FCS-based techniques have a unique status in this regard, and they have been utilized for elucidating microsecond dynamics of proteins. In fact, it has been shown that intensity-based FCS is able to detect conformational dynamics of polypeptides with a time resolution down to nanoseconds^17^,^41^. A problem of the conventional FCS is the difficulty in making clear assignments of observed kinetics to relevant substates (see Fig. 2c). Therefore, when the system becomes complex, one needs to rely on the theoretical modelling for interpretation^42^. Separation of the substates contributing to the FCS signal is greatly facilitated by the use of fluorescence lifetime information. Enderlein *et al*.^43^,^44^ developed a systematic way to incorporate fluorescence lifetime measurements to FCS, and their method is capable of decomposing the FCS curve into the auto and cross-correlations of all substates referring to the authentic fluorescence decay curve of each substate. Although this technique provides a good basis to examine well-characterized systems^45^, the necessity of the reference decay curves seriously limits its applications. In this work, it is demonstrated that 2D FLCS is capable of determining the number of distinct substates, the fluorescence decay curve of each substate and the interconversion rates between individual substates from a photon data set without any *a priori* information. 2D FLCS allows visualization of complex conformational dynamics of proteins in equilibrium on a wide range of timescales.

In summary, the present 2D FLCS study unveils the presence of several conformers in each conformational ensemble and clarifies their transitions on the microsecond timescales, using rigorous quantitative analysis based on the fluorescence lifetime correlation. This provides new insights into the complex free energy landscape of proteins. Elucidation of the hierarchical conformational dynamics of proteins is essential for understanding their functions, and the dynamics on the microsecond timescale are the key, because direct comparison between experiment and simulation is possible^7^. Thus, 2D FLCS will be a very powerful tool to study the structural dynamics as well as relevant functions of proteins.

## Methods

### Sample preparation

Cytochrome *c* from *Saccharomyces cerevisiae* (cyt *c*) was purchased from Sigma-Aldrich (C2436) and Alexa546 C5 maleimide was purchased from Life Technologies (A-10258). Other reagents were of analytical grades and used without any purifications. Cyt *c* was applied to the cation-exchange column (GE Healthcare SOURCE 15S 4.6/100 PE) for purification. Alexa546 was attached to the single free cysteine residue of cyt *c* by using the protocol provided by the manufacturer. Oxidation of cyt *c* was done by incubating cyt *c* in 10 mM Tris-HCl buffer, pH 7.4, containing potassium ferricyanide (final conc. 10 mM) for 10 min. Potassium ferricyanide was then removed by size-exclusion chromatography. For reference measurements, Alexa546 was reacted with an excess amount of DTT in 20 mM Tris-HCl buffer for 2 h. The reaction of the maleimide group with DTT was required because Alexa546 with unreacted maleimide showed a biexponential fluorescence decay and also showed inhomogeneity in lifetime-weighted FCS. This is probably due to the photo-induced electron transfer from dye to maleimide as was reported for the fluorescence of BODIPY^46^. For the measurements with pH change, the sample was diluted with the following buffer: glycine-HCl buffer, pH 1.0–3.0; acetate buffer, pH 3.5–5.5. The buffer concentration was kept at 100 mM.

### FCS and fluorescence lifetime measurements

FCS measurements were performed on a home-built correlation spectrometer based on a femtosecond Ti:sapphire oscillator (Coherent Mira 900-F) and an inverted microscope (Nikon TE-2000U). The detail of the instrument has been described elsewhere^28^. Briefly, an optical parametric oscillator (Coherent Mira-OPO) was used to generate 540 nm excitation pulses from the 775 nm output of the Ti:sapphire oscillator. The excitation power was adjusted to∼40 μW at the entrance of the microscope. The fluorescence signal from the sample was collected with the epifluorescence geometry and separated from the excitation path by a dichroic mirror. Then, it was passed through a confocal pinhole and an optical filter (Chroma Technology D585/40 m), and was split into two by a nonpolarizing beam splitter before detection by two photon-counting avalanche photodiodes (id Quantique id 100-20). The full width at half maximum of the instrumental response was ∼50 ps. For each detected photon, two types of temporal information, macrotime and microtime, were recorded by a TCSPC module (Becker & Hickl SPC 140) and stored as photon data. Macrotime is the absolute photon arrival time from the start of the experiment, and microtime is the relative delay time between the excitation pulse and detected fluorescence signal (referred to as emission delay time). Cross-correlation of the photon data obtained with two detectors were calculated and analysed to avoid the artifact due to the after-pulsing effect of the detector. The sample cell consisted of a silicone spacer sandwiched by two cover slips. To avoid the sample adsorption on the surface of the cover slip, the sample cell was filled with unlabelled cyt *c* solution (20 μM) and incubated for 1 h for coating^25^. The unlabelled cyt *c* solution was removed from the cell just before adding the sample solution. The sample concentration was set at ∼10 nM.

### Lifetime-weighted FCS

Lifetime-weighted FCS can detect the inhomogeneity of the sample and its time evolution^28^. The ordinary fluorescence intensity correlation function (*G*_I_) and lifetime-weighted fluorescence correlation function (*G*_L_) are calculated from photon data as follows^28^:


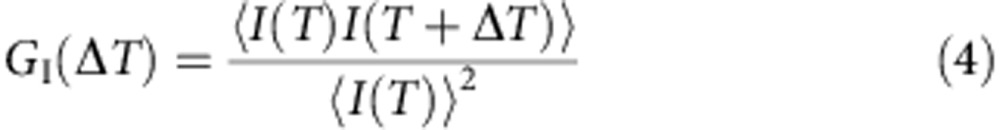



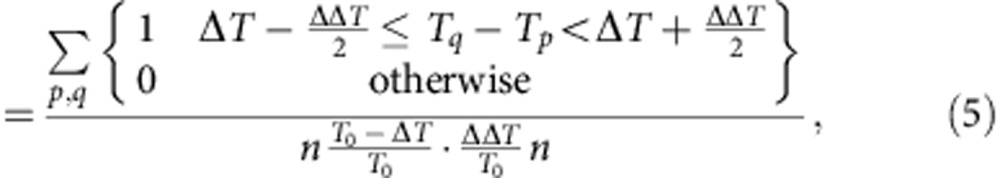



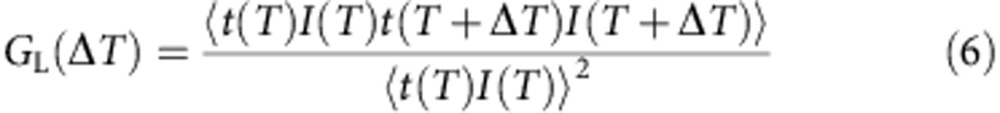



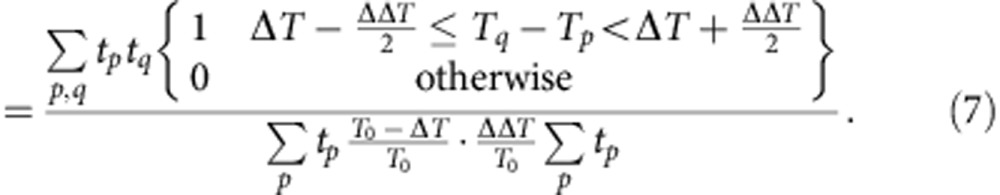


In equations (4)–(7), 5, 6, 7, *T*_*i*_ is the macrotime of the *i*th photon, Δ*T* is the macrotime delay, ΔΔ*T* is the width of the temporal window, *T*_0_ is the total measurement time, *n* is the total number of photons detected during the measurement and *t*_*i*_ is the microtime of the *i*th photon. Calculation was performed by a code written in C and was done by Igor Pro (Wavemetrics).

The correlation ratio (*G*_R_) between *G*_L_ and *G*_I_ is calculated according to equation (8).


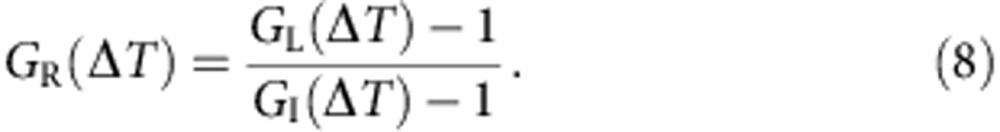


### 2D fluorescence lifetime correlation spectroscopy

2D FLCS was performed based on the method reported by Ishii and Tahara^18^,^19^,^20^. Briefly, a 2D emission-delay correlation map for a certain macrotime delay Δ*T* 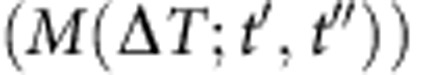 was first generated from the photon data consisting of the macrotime and microtime of each photon. Then, the correlated part 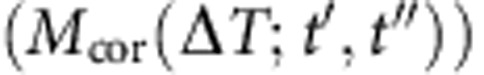 was obtained by subtracting the uncorrelated part 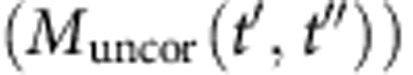:


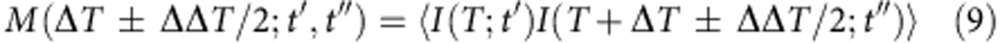



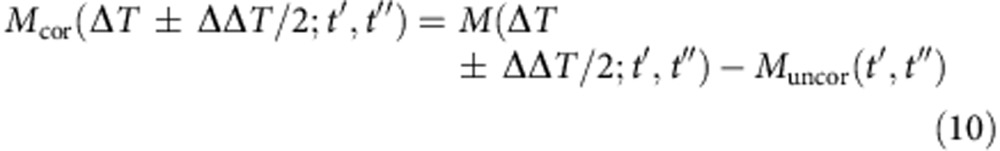



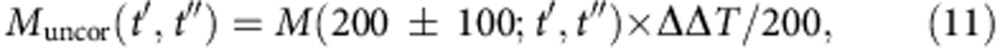


where ΔΔ*T* is the temporal window size at Δ*T*. We used the 2D emission-delay correlation map at Δ*T*=200±100 ms as the uncorrelated part of the 2D map because the correlation is practically lost at a very large Δ*T* due to diffusion. For the obtained 2D emission-delay correlation map 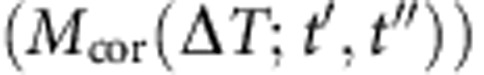, 2D ILT was performed with the help of maximum entropy method (MEM). ILT converts the coordinates of the map from the emission delay time (*t*) to the fluorescence lifetime (*τ*), and hence converts the 2D emission-delay correlation map 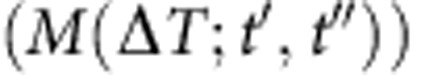 to 2D lifetime correlation map 
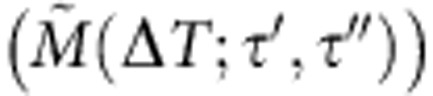
. We note that MEM is needed in this conversion to suppress the numerical instability of ILT. We describe the essential part of 2D MEM analysis in the following, although the details have been already reported in our previous paper^19^,^20^.

Because the correlated part of the emission-delay correlation map 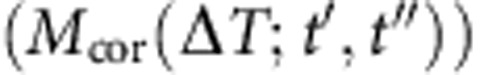 is the sum of the single-molecule correlation, 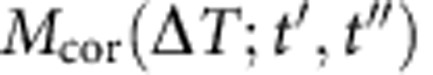 can be represented as,


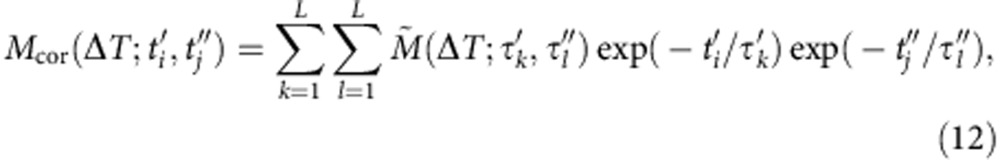



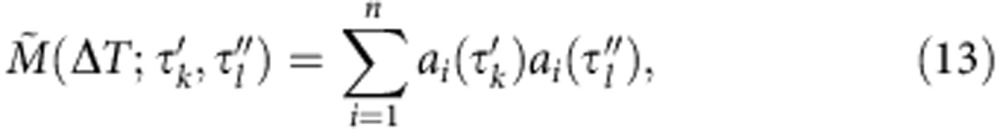


where *a*_*i*_(*τ*) is the lifetime distribution of species *i*. Then, a trial 2D lifetime distribution, 
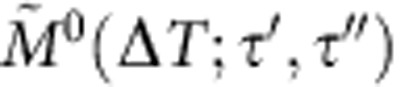
, is set to calculate a simulated 2D emission-delay correlation map:


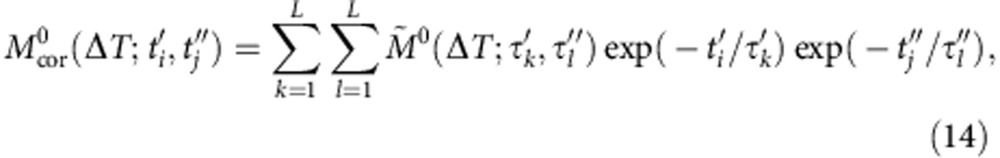



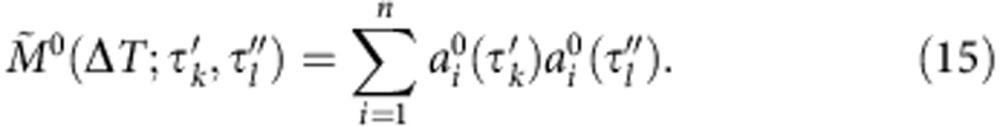


The fitting error (*χ*^2^) and the entropy (*S*) of the 2D lifetime correlation map can be defined as


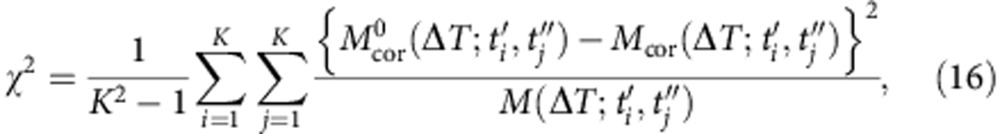



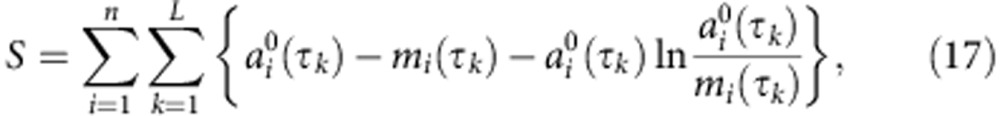


where *m*_*i*_(*τ*) is *a priori* knowledge of 
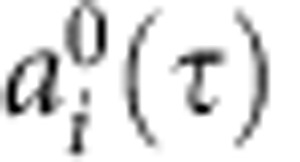
. In the present case, it is reasonable to think that the ensemble-averaged fluorescence decay curve and the corresponding fluorescence lifetime distribution shown in Fig. 2b contain all the lifetime components of correlated species. Therefore, the fluorescence lifetime distribution shown in the inset of Fig. 2b was used for *m*_*i*_(*τ*) as *a priori* information in this analysis. This *m*_*i*_(*τ*) value acts as a bias to determine each lifetime distribution forming the 2D lifetime correlation map and suppresses the numerical instability of ILT. Then, the optimum distribution 
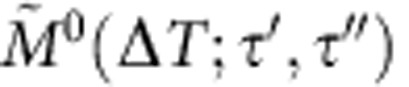
 that minimizes the following *Q* value is searched and determined,


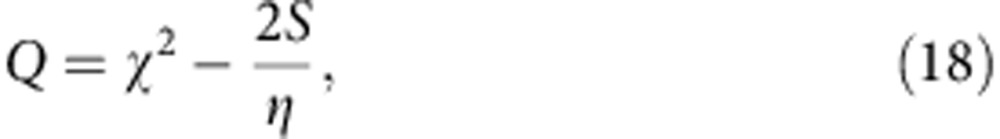


where *η* is the regularizing constant.

In actual analysis, the microtime resolution was reduced from 4,096 (3 ps per channel) to 256 (48 ps per channel) channels by binning 16 adjacent data points into a single channel (4,096 is the total available time channel of the TCSPC module used in this study). Then, the 2D emission-delay correlation map (256 × 256 channels) was constructed with reduced microtime resolution (shown in Fig. 3a–c). For 2D MEM analysis, the microtime range from 0 to 6.14 ns (corresponds to 128 × 128 channels) was selected. The microtime resolution of the selected 2D emission-delay correlation map was further reduced to 24 × 24 channels in the analysis to save computation time. In this procedure, the binning width was changed logarithmically along the microtime axes to keep the lifetime information as much as possible. Discrete *τ* values (total 40 *τ* points) that are equally distributed in a logarithmic scale between 0.05 and 10 ns were used for determining *a*_*i*_(*τ*) and 
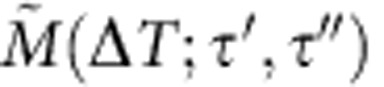
. For 2D lifetime correlation maps shown in Fig. 3d–f in the main text and Supplementary Fig. 4, each 2D map was interpolated using spline curve for the visual purpose (The final 2D maps consist of 201 × 201 channels).

For the 2D map calculated for Δ*T*=0.2–4 μs, ILT with MEM was performed by changing the number of independent species (*n*) from 2 to 5. Up to *n*=4, each lifetime distribution (*a*_*i*_(*τ*)) changed with the change of *n* and exhibited unique peak pattern (data not shown). In case of *n*=5, however, two of the lifetime distributions showed an identical peak pattern, indicating that four independent species are necessary and sufficient to reproduce the 2D emission-delay correlation map at Δ*T*=0.2–4 μs. The four independent lifetime distributions were determined in this way. For the 2D map calculated for Δ*T*=8–12 μs, two of the lifetime distributions showed identical lifetime distribution, indicating that three independent species are enough to describe the 2D emission-delay correlation maps at Δ*T*>10 μs. Therefore, *n*=3 was set to analyse the 2D maps calculated for Δ*T*>10 μs.

### The global analysis of fluorescence decay curves

Four independent lifetime distributions were identified by 2D fluorescence lifetime correlation analysis (sp1∼sp4 in Fig. 3g) as described above. For the global analysis of pH-dependent fluorescence decay curves, these lifetime distributions were used. However, although five distinct fluorescence lifetimes appear in sp1∼sp4 in total, we only used four (*τ*_1_=70 ps, *τ*_2_=280 ps, *τ*_3_=1.7 ns, *τ*_4_=3.3 ns) for the analysis of pH-dependent fluorescence decay curves because the lifetimes corresponding to the peak in sp2 and the first peak in sp3 are very close to each other. Thus, the lifetime corresponding to the single peak of sp2 (*τ*_2_=280 ps) was used as the representative of those two peaks in the analysis. Then, pH- and guanidium hydrochloride-dependent fluorescence decay curves shown in Supplementary Fig. 2a were fitted using the following equation,


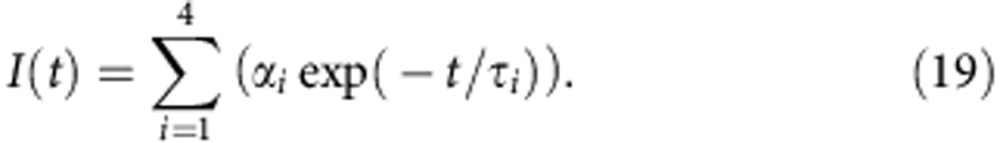


In the fitting, the four fluorescence lifetime values were fixed, and only amplitudes of the components (*α*_*i*_) were adjusted to fit each decay curve. Fitting results and the amplitudes of each lifetime component are shown in Supplementary Fig. 2a,b, respectively.

### Fitting of auto and cross-correlation of each substate

The fitting analysis for the autocorrelations of N (*G*_NN_) and I_1_ (*G*_II_) and that for cross-correlation between N and I_1_ (*G*_NI_) were performed with the following functions based on the two-state equilibration system^45^:


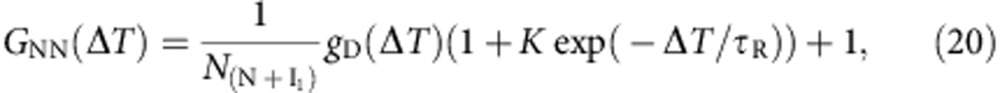



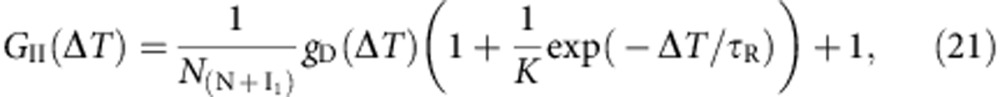



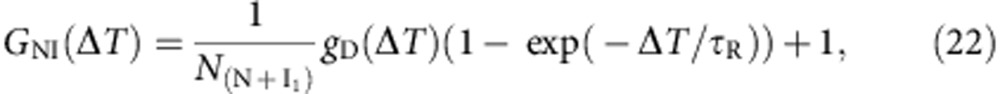



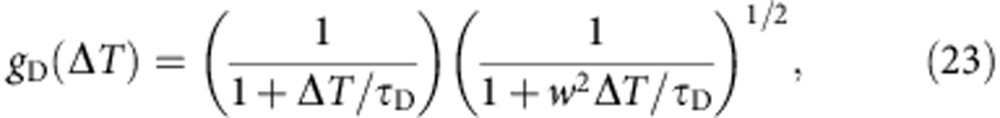



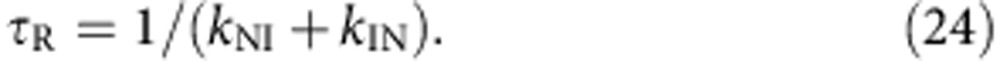


In equations (20)–(24), 33, 34, 35, 36, *N*_(N+I1)_ is the sum of the average number of N and I_1_ molecules in the focal region, *g*_D_ (Δ*T*) is the correlation due to translational diffusion of a molecule into and out of the focal region, 
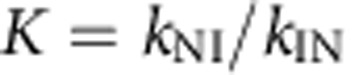
 is the equilibrium constant between N and I_1_, *τ*_R_ is the conformational equilibration time between N and I_1_, *τ*_D_ is the diffusion time constant, *w* is the radial-axial ratio in the focal region and *k*_NI_ and *k*_IN_ are the conformational transition rate constant from N to I_1_ and from I_1_ to N, respectively. An equilibrium constant (*K*) was determined by taking the intensity ratio of the diagonal peaks between N and I_1_ in 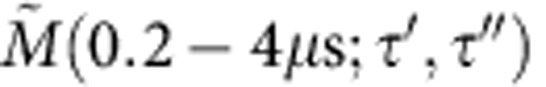 and the radial-axial ratio was determined by fitting the correlation curve of Alexa546_DTT in buffer solution with the theoretical equation (equation (25) shown below). The obtained *K* (=0.25) and *w* (=0.078) values were used in the fitting for the correlation functions of N and I_1_.

For the fitting of autocorrelations of I_en_ and U, two theoretical formulas described by equations (25) and (26) were tested:


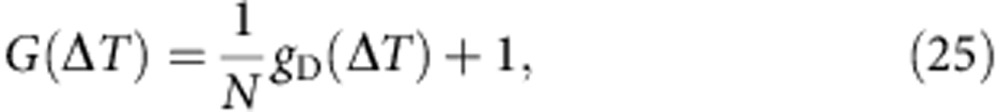



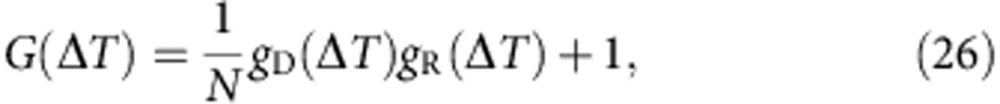



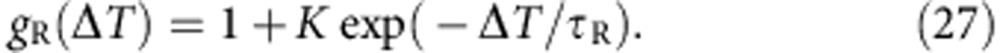


Equation (25) only contains a contribution from the translational diffusion (*g*_D_ (Δ*T*)), and equation (26) contains a contribution from the conformational equilibration (*g*_R_ (Δ*T*)) in addition to that from the translational diffusion. In the fitting using equation (25), the fitting region was limited to 10 μs–1 ms, where the correlation due to translational diffusion is predominant.

### Derivation of fluorescence correlation functions

The adequacy of the proposed scheme (Fig. 6) was checked by calculating the ordinary fluorescence intensity correlation and the lifetime-weighted fluorescence correlation shown in Supplementary Fig. 1. The calculated curves reproduced the experimental data very well, confirming the adequacy of Scheme 2. The details of the analysis are described in the following.

For the time region of 1 μs<Δ*T*<1 ms, Scheme 2 in Fig. 6 can be rewritten as three sets of two-state equilibration system, that is, N↔I_1_, I_en_↔D_1_ and U↔D_2_, where I_en_ represents I_2_ and I_3_ which are in a fast equilibrium. In this case, the correlation function can be described as follows, assuming that the diffusion time constant are the same for all the substates:


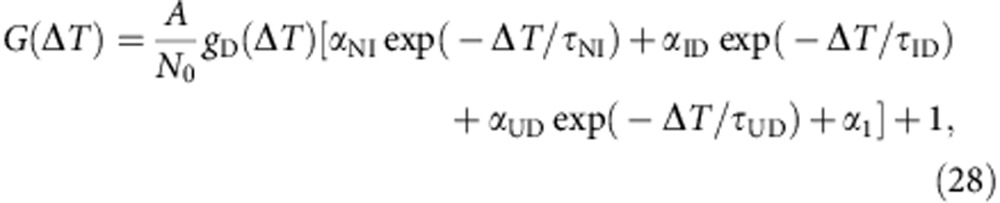



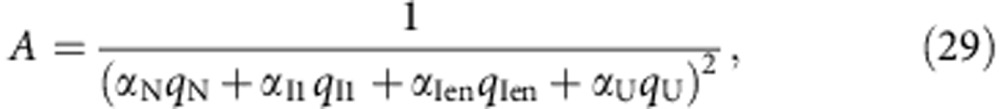



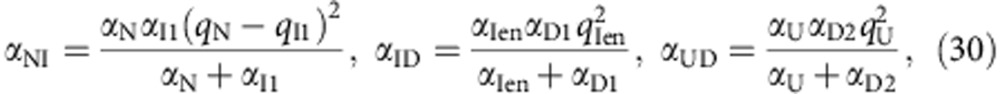



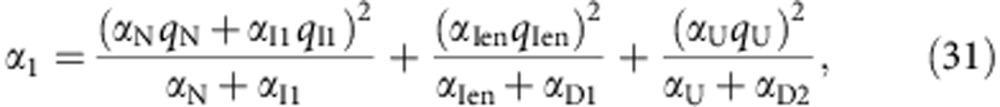



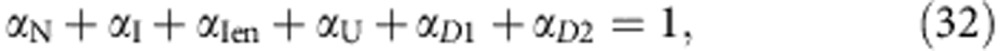



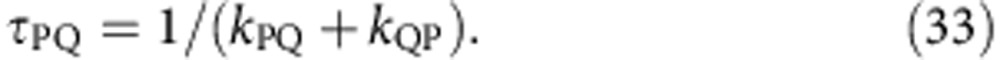


Here *N*_0_ is the average number of all molecules in the focus region, *q*_P_ and *α*_P_ are the brightness and relative population of substate P, respectively, and *τ*_PQ_ is the conformational equilibration time between substates P and Q. The brightness of D_1_ and D_2_ were set at 0. In the case of lifetime-weighted correlation, *q*_P_ is replaced with *τ*_P_*q*_P_, where *τ*_P_ is the fluorescence lifetime of substate P.

For equation (28), the relative population of each substate (*α*_P_) except for D_1_ and D_2_ can be determined from the fitting of the fluorescence decay curve at pH 3.5. For that purpose, Laplace transform was performed on the independent lifetime distributions (sp1∼sp4, shown in Fig. 3g), and the corresponding fluorescence decay curves were obtained. The fluorescence amplitude at the time origin of component was normalized to 1 before convoluting with the instrumental response function. Then, the obtained decay curves of the four independent lifetime distributions (*I*_P_ (*t*); P=1–4) were used to fit the fluorescence decay curve at pH 3.5 (Fig. 2b), and the relative populations (*α*_P_) of each lifetime distribution were obtained (Supplementary Fig. 7b). *I*_P_ (*t*)'s were also used for determining the effective *τ*_P_ (fluorescence lifetime) and relative *q*_P_ (brightness) of each lifetime distribution in the following way. In the actual experiment, microtime information of each photon was collected only in the range of 0 ns<*t*<9.5 ns, which does not cover the whole range of the decay curve, in particular, for long lifetime components. Thus, *I*_P_(*t*) was truncated to meet the microtime detection window, and *τ*_P_ and *q*_P_ were determined from the following truncated form of *I*_P_(*t*) assuming the extinction coefficient of Alexa546 is the same irrespective of protein conformations:


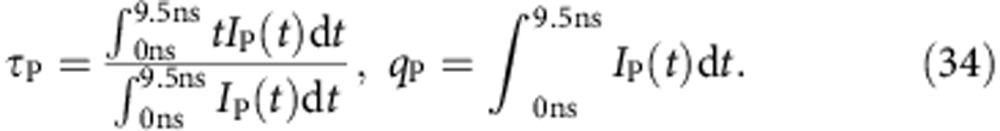


The obtained *α*_P_, *τ*_P_ and *q*_P_ are listed in Supplementary Fig. 7b.

With the procedure described above, the *α*_P_, *τ*_P_ and *q*_P_ values in equation (28) were fixed. The conformational equilibration times between N and I_1_ (*τ*_IN_=5 μs), I_en_ and D_1_ (*τ*_ID_=7 μs), and U and D_2_ (*τ*_UD_=4 μs) have been determined from the fitting of autocorrelations and cross-correlation shown in Fig. 5. The radial-axial ratio *w*=0.078 was determined by the fitting of the correlation curve of Alexa546_DTT. With these values fixed, we carried out fitting analysis for ordinary fluorescence intensity correlation and lifetime-weighted fluorescence correlation using equation (28). Adjustable parameters are *N*_0_, *α*_D1_, *α*_D2_ and *τ*_D_. The best fits successfully reproduced the experimental correlation curves (Supplementary Fig. 7a). The parameters determined by the fitting are listed in Supplementary Fig. 7c. It is worth noting that the calculated correlation curves exhibit subtle wavy features which are not recognized in the experimental data. This small deviation was completely vanished when the stretched exponential function was used to describe the conformational equilibration dynamics between I_en_ and D_1_ as well as that between U and D_2_ (data not shown). This may suggest that the equilibration time between these substates has a distribution. However, this deviation is small so that the essence of the argument of this study is not affected.

## Additional information

**How to cite this article:** Otosu, T. *et al*. Microsecond protein dynamics observed at the single-molecule level. *Nat. Commun.* 6:7685 doi: 10.1038/ncomms8685 (2015).

## Supplementary Material

Supplementary Information Supplementary Figures 1-7, Supplementary Notes 1-5 and Supplementary References.
