# Supplementary material for: Microsecond protein dynamics observed at the single-molecule level
Source: Nat Commun. 2015 Jul 7;6:7685. doi: 10.1038/ncomms8685 (PMC4506535; doi:10.1038/ncomms8685)
Supplement: Supplementary Information — Supplementary Figures 1-7, Supplementary Notes 1-5 and Supplementary References. [file ncomms8685-s1.pdf]

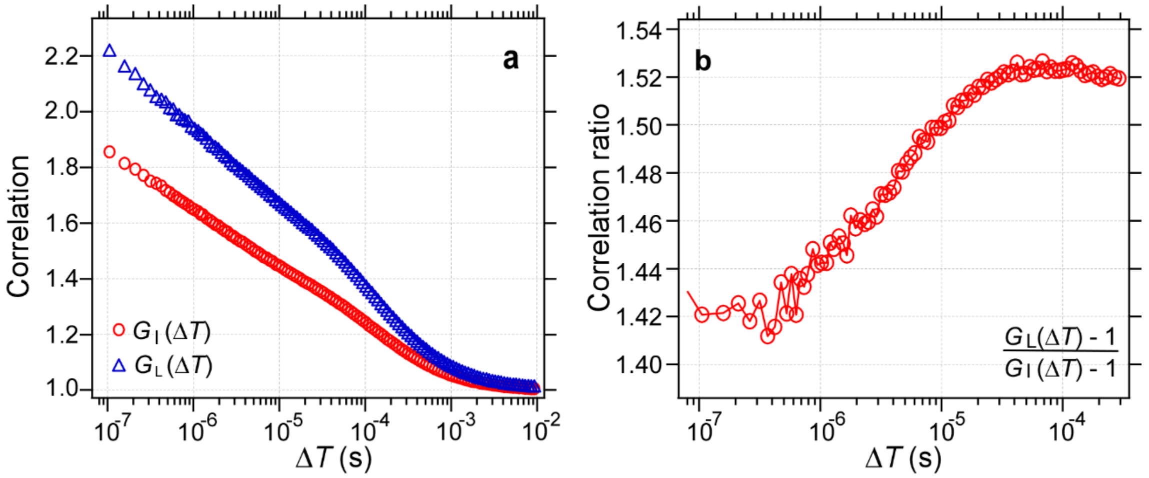

Supplementary Figure 1

Lifetime-weighted FCS.

(a) The fluorescence intensity correlation (red open circle, $G_I(\Delta T)$) and the lifetime-weighted correlation (blue open triangle, $G_L(\Delta T)$) of Alexa546_cytochrome *c* at pH3.5. **(b)** The ratio of the lifetime-weighted correlation to the intensity correlation. The ratio was calculated by the equation shown in the figure.

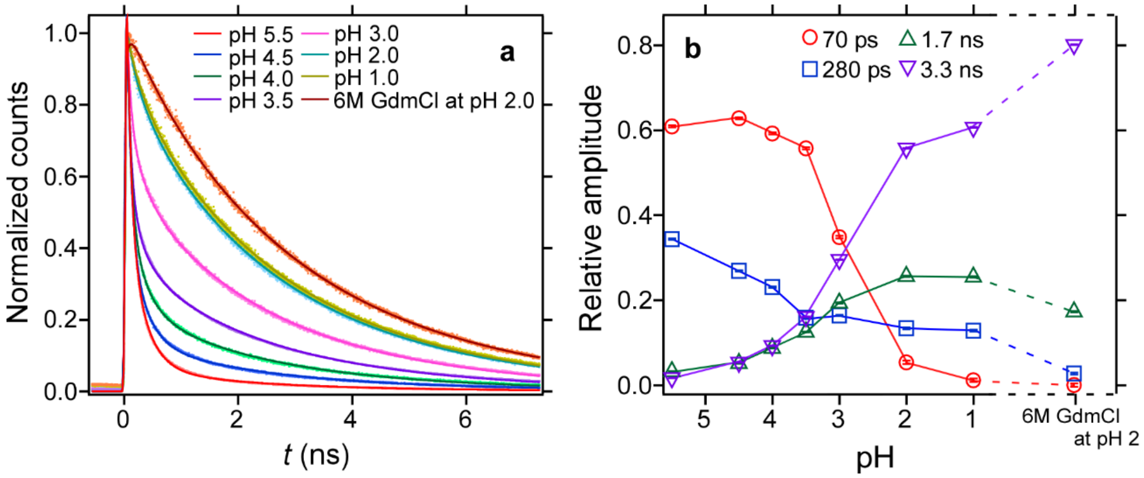

Supplementary Figure 2

The result of global fitting.

(a) pH- and denaturant-dependent fluorescence decay curves of Alexa546_cytochrome *c* (dot). The data are fitted using four fluorescence lifetimes shown in **b**, and the fitting results are shown by solid lines with corresponding colors. Data are normalized by the intensity at t = 0. Solvent conditions of each decay curve are listed in the figure. **(b)** Relative amplitudes of each lifetime component (the lifetimes are shown in the figure) obtained by global fitting of pH- and denaturant-dependence of fluorescence decay curves shown in **a.**

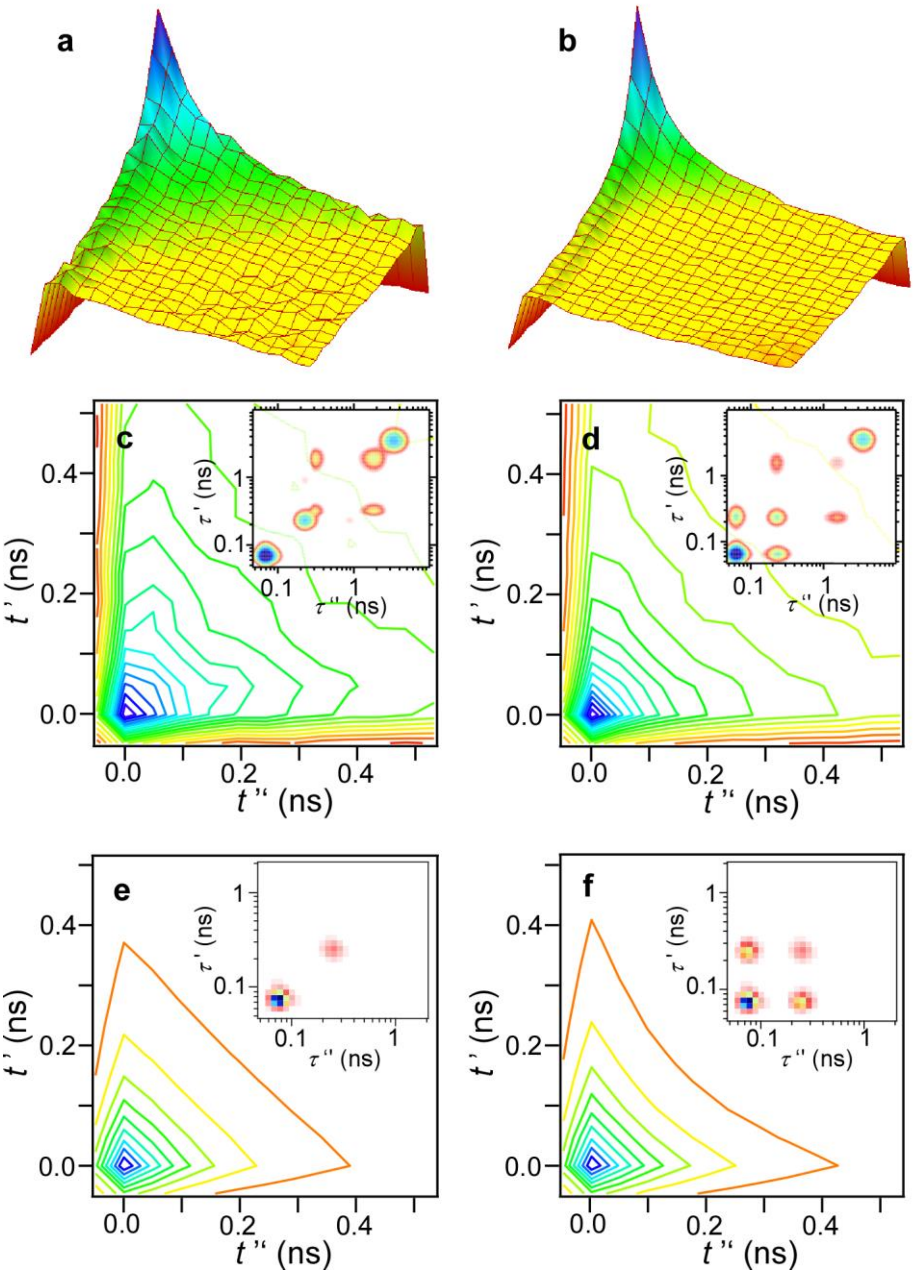

Supplementary Figure 3

2D emission-delay correlation maps.

The image (**a**,**b**) and the contour (**c**,**d**) plots of 2D emission-delay correlation maps calculated at ΔT = 0.2 – 4 μs (**a**,**c**) and ΔT = 50-100 μs (**b**,**d**) are shown. The regions t = 0 ~ 0.92 ns (**a**,**b**) and t = -0.05 ~ 0.54 ns (**c**,**d**) are shown. The simulated 2D emission-delay correlation maps (**e**,**f**) are calculated by Laplace transform of simulated 2D lifetime correlation maps shown in the inset.

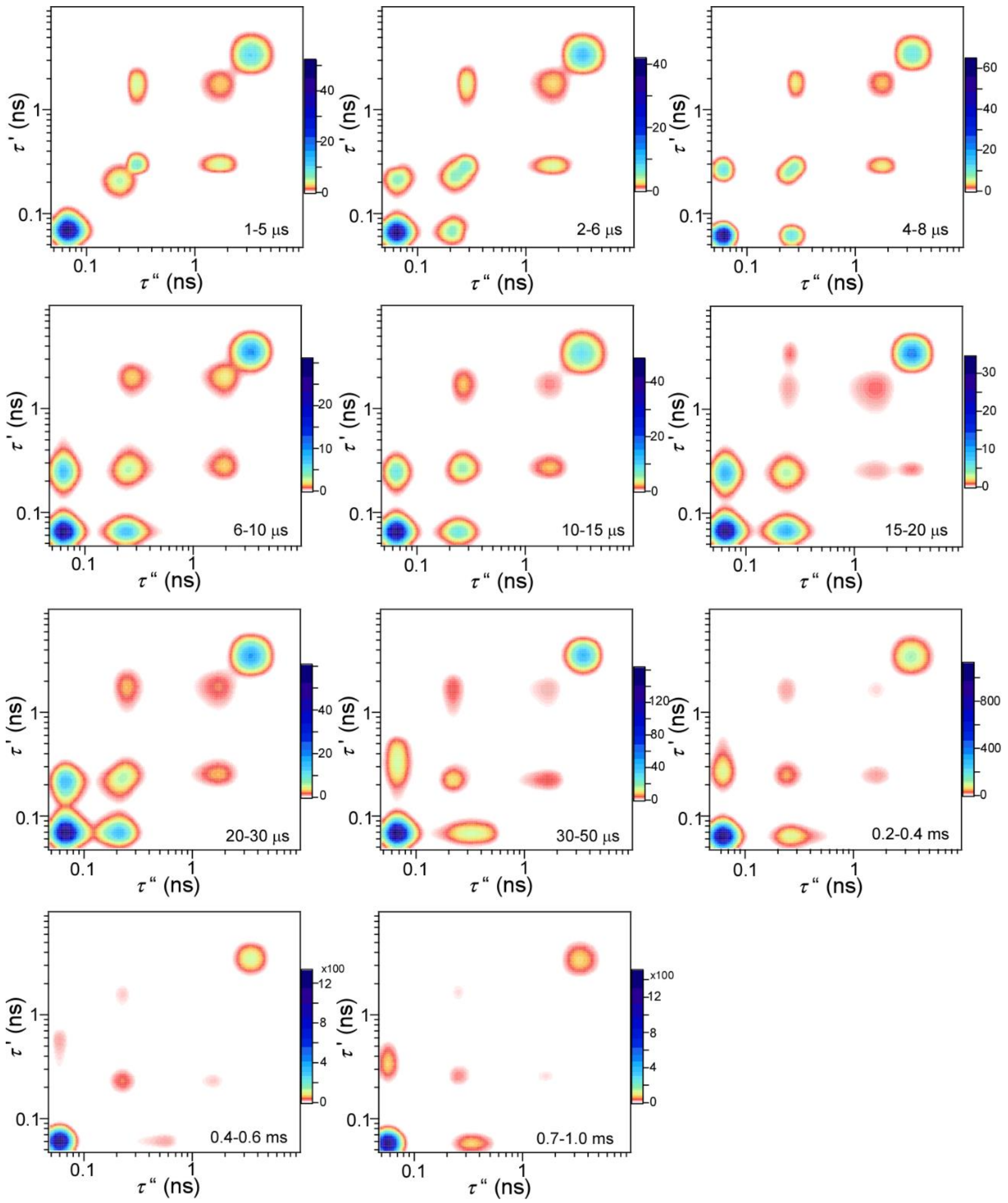

Supplementary Figure 4

2D lifetime correlation maps calculated at different Δ*T*.

The time widths of Δ*T* are shown in the figure. The data are smoothed using spline curves for visual purpose.

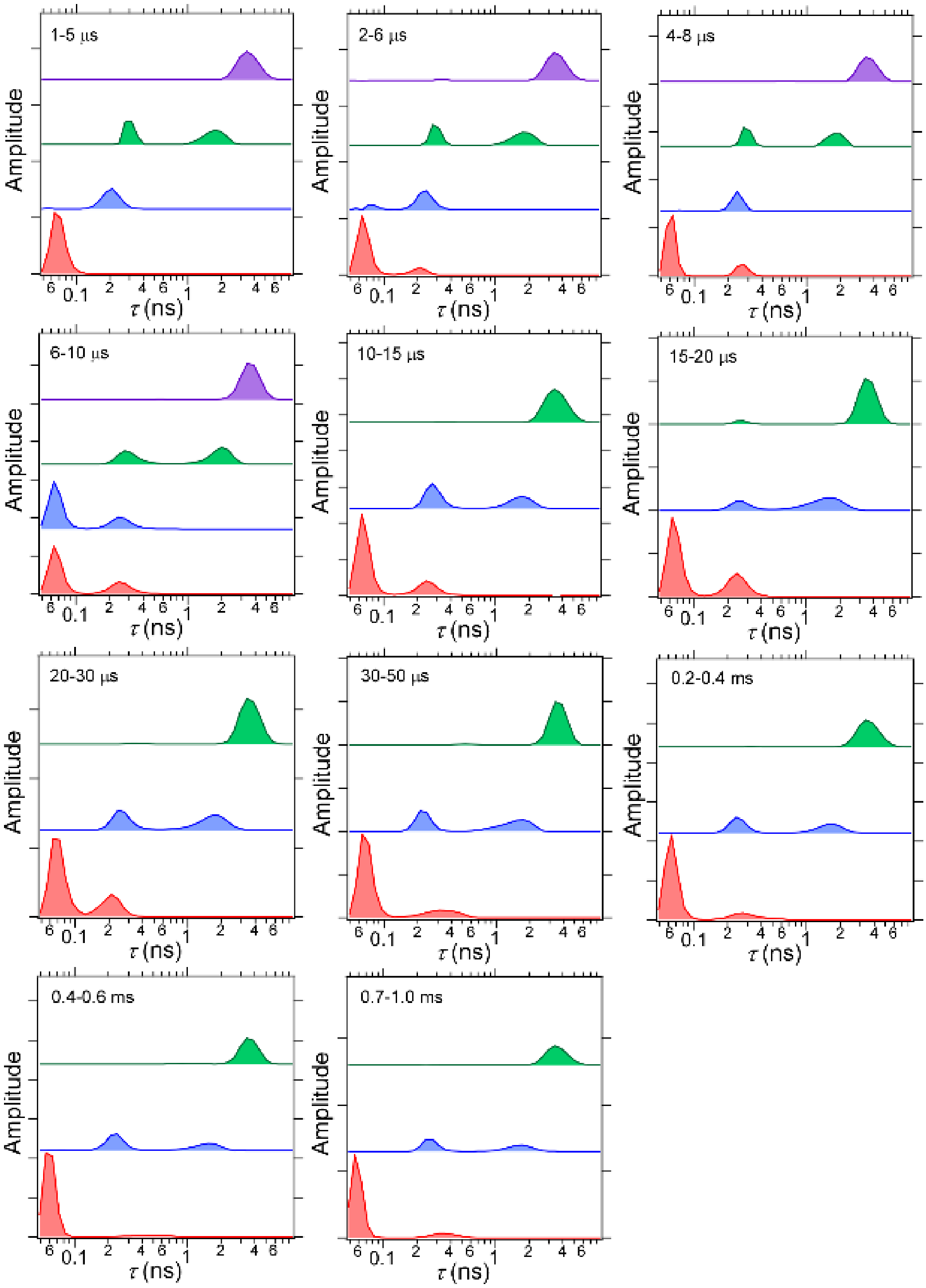

Supplementary Figure 5

Independent lifetime distributions calculated at different Δ*T*.

The time widths of Δ*T* are shown in the figure.

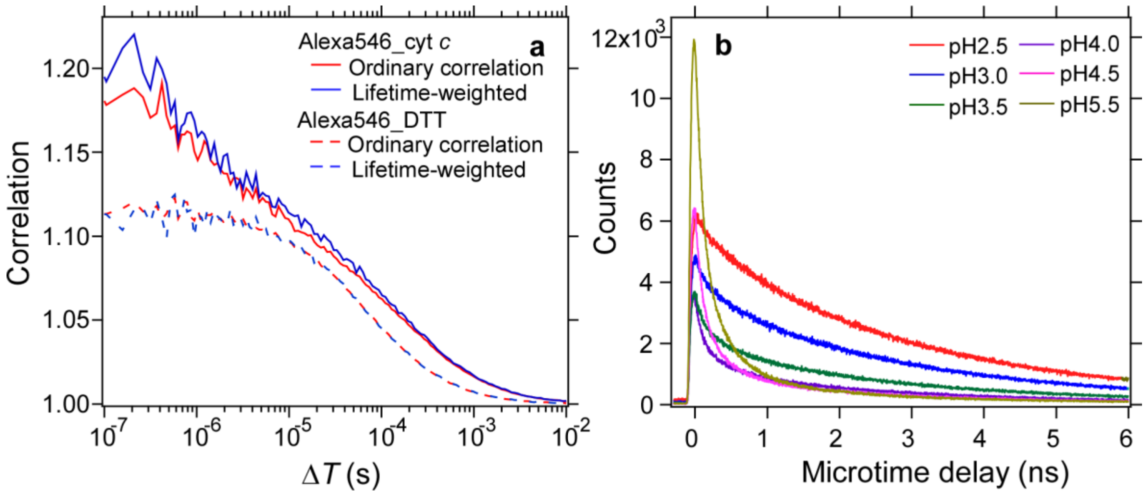

Supplementary Figure 6

Lifetime-weighted FCS and pH-dependent fluorescence decay curves.

(a) The fluorescence intensity (red) and lifetime-weighted (blue) correlation curves of Alexa546_cytochrome *c* (solid line) and Alexa546 reacted with DTT (broken line) measured at pH 1.0. **(b)** Un-normalized fluorescence decay curves of Alexa546_cytochrome *c* at various pH conditions.

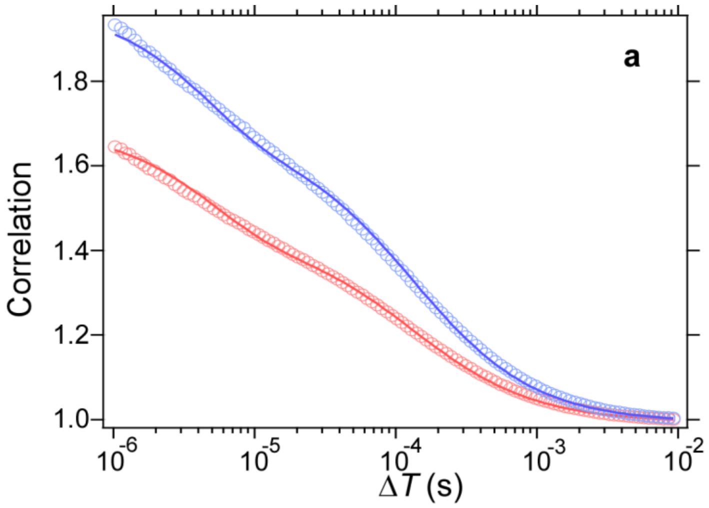

b

	α_p	τ_p (ns)	q_p
N	0.51	0.07	0.07
I_1	0.13	0.40	0.27
$^*I_{en}$	0.22	1.75	1.27
U	0.14	2.86	3.02

$^*I_{en} = I_2 + I_3$

c

α_{D1}	0.54 ± 0.05	N_0	6.6 ± 0.2
α_{D2}	0.05 ± 0.00	τ_R [N $\rightleftharpoons$ I_1] (μs)	*5.3
τ_D (μs)	130 ± 1.2	τ_R [I_{en} $\rightleftharpoons$ D_1] (μs)	*7.0
w	*0.078	τ_R [U $\rightleftharpoons$ D_2] (μs)	*4.0

* the values are fixed during the fitting

Supplementary Figure 7

Comparison between experimental and theoretical correlation curves.

(a) Experimental (open circle) and theoretical (solid line) fluorescence intensity correlation (red) and fluorescence lifetime-weighted correlation (blue) curves **(b)** Table showing the relative population (α_p), the fluorescence lifetime (τ_P), and the relative brightness (q_P) of each substate. **(c)** Table showing the fitting results. α_{D1} and α_{D2} are the relative value with the total population of emissive species being 1.

Supplementary Note 1. Assignment of the peaks in the 2D lifetime correlation map: Assignment of each diagonal peak in the 2D lifetime correlation map was made based on the pH and denaturant dependences of the fluorescence decay. Supplementary Figure 2 shows the full set of pH- and denaturant-dependent fluorescence decay data of Alexa546_cyt *c* measured in this study. As readily seen, the 70-ps lifetime component has the largest contribution at pH 5.5 where the native state is predominant. On the other hand, the 3-ns lifetime component is predominant in the data at pH 1.0 as well as those under a high denaturant condition in which cyt *c* is denatured. Therefore, it is reasonable to assign 70-ps (sp1) and 3-ns (sp4) lifetime components to the native state (N) and the unfolded state (U) of cyt *c*, respectively. As for the 280-ps and 1.7-ns components, the fluorescence decay under a high denaturant condition contains almost negligible contribution from the 280-ps lifetime component but substantial contribution from the 1.7-ns lifetime component. This implies the two distinct fluorescence lifetimes of sp3 do not stem from a single conformation that shows a biexponential decay, but result from two distinct emitting species. In other words, this result indicates that the equilibration process (i.e., conformational transition) between the substates exhibiting two fluorescence lifetimes in *sp3* occurs within 1 μs. Therefore, the peaks in sp2 and sp3 can be assigned to three different folding intermediate states, that is, I_1 (sp2), I_2 (shorter lifetime component of sp3), and I_3 (longer lifetime component of sp3).

Supplementary Note 2. Equilibration process between N and I_1 recognized in the 2D emission-delay correlation map:

The 2D lifetime correlation maps shown in Fig. 3d-f clearly show the equilibration process between N and I_1 as an appearance of the cross peaks. This equilibration process can also be recognized in the 2D emission-delay correlation maps before ILT.

Supplementary Figures 3a and b show the 2D emission-delay correlation maps at ΔT = 0.2 - 4 μs and ΔT = 50-100 μs, respectively, for the microtime region of t = 0 ~ 0.93 ns. The corresponding contour plots are also shown in Supplementary Figs. 3c and d for the microtime region of t = 0 ~ 0.5 ns. The key difference is that a "ridge" appears along the zero-time line in the 2D map at ΔT = 50 - 100 μs (Supplementary Figs. 3b and 3d) whereas it is missing in the 2D map at ΔT = 0.2 - 4 μs. This ridge is a typical feature that arises from the correlation between the short lifetime and long lifetime components[1,2]. Because the experimental 2D emission-delay correlation maps also contain contributions other than those from N and I_1, we simulated 2D maps assuming that only N and I_1 exists in the system to confirm that the above-mentioned difference surely reflect the equilibration process between N and I_1. In this simulation, we first simulated 2D lifetime correlation maps for the two cases: one is the case that the equilibration process occurs slower than ΔT (inset of Supplementary Fig. 3e), and the other is the case that the equilibration occurs faster (inset of Supplementary Fig. 3f). The relative population between the 70-ps and 280-ps lifetime components as well as the width of their peaks was set at the same values as those in the experimental data (sp1 and sp2 in Fig. 3d in main text). Then, the simulated 2D lifetime correlation maps were converted to 2D emission-delay correlation maps by Laplace transform. As seen in Supplementary Figs. 3e and f, the simulated 2D emission-delay correlation maps reproduced essential features of the experimental 2D emission-delay correlation maps (i.e., the presence and

absence of the ridge along the zero time line). This confirms that the equilibration process between N and I_1 is indeed recognizable even in the experimental 2D emission-delay correlation map. This also confirms the reliability of ILT using the 2D maximum entropy method to extract correct information about the fluorescence lifetime from experimental data.

Supplementary Note 3. Existence of I_1 and I_2:

The I_1 and I_2 states give two distinct peaks at 280 ps and 300 ps in the 2D lifetime correlation maps at ΔT = 0.2-4 μs (Fig. 3d), whereas they are converged and become indistinguishable in the 2D maps at longer delay times (Figs. 3e and 3f). This is due to the limited accuracy of lifetime determination of the MEM-based analysis. However, this convergence does not affect our argument about the existence of the I_1 and I_2 states that have fluorescence lifetimes of ~300-ps. As clearly seen in the 2D map for ΔT longer than 10 μs, the cross peaks between 70 ps and 280 ps and those between 280 ps and 1.7 ns are clearly observed. On the other hand, no cross peak is observed between 70 ps and 1.7 ns components. This feature cannot be rationalized with a single state having ~300-ps lifetime because, if so, the cross peak between 70 ps and 1.7 ns must be observed. Thus, we can conclude that there are two independent states having ~ 300-ps lifetimes, and one state (I_1) is in equilibrium with N (70 ps) state while another (I_2) is in equilibrium with I_3 (1.7 ns) state, within 10 μs.

Supplementary Note 4. Autocorrelation and cross-correlation of each substate:

Fluorescence autocorrelation functions of N, I_1, I_{en}, and U as well as the cross-correlation function between N and I_1 were obtained from $\tilde{M}(\Delta T;\tau',\tau'')$ or using independent lifetime distributions $\alpha(\Delta T;\tau)$. After an explanation about the concept of the component-specific correlation function, the actual procedure is described.

1. Concept of component-specific correlation function

The relationship among the ordinary fluorescence intensity correlation function ($G_{\mathrm{I}}(\Delta T)$), 2D emission-delay correlation map ($M(\Delta T;t',t'')$) and 2D lifetime correlation map ($\tilde{M}(\Delta T;\tau',\tau'')$) is written as follows[1]:

$$G_{\mathrm{I}}(\Delta T) = \frac{\iint M_{\mathrm{cor}}(\Delta T;t',t'')dt'dt''}{\iint M_{\mathrm{uncor}}(t',t'')dt'dt''} + 1$$

$$= \frac{\iint\iint \tilde{M}_{\mathrm{cor}}(\Delta T;\tau',\tau'')\exp(-t'/\tau')\exp(-t''/\tau'')d\tau' d\tau'' dt' dt''}{\iint\iint \tilde{M}_{\mathrm{uncor}}(\tau',\tau'')\exp(-t'/\tau')\exp(-t''/\tau'')d\tau' d\tau'' dt' dt''} + 1 \quad . \qquad (1)$$

When the sample contains different fluorescent species and the observed fluorescence decay consists of multiple fluorescence lifetime components, the component-specific correlation function ($G_{\mathrm{I};i,j}(\Delta T)$) can be written in an analogous way:

$$G_{\mathrm{I};i,j}(\Delta T) = \frac{\iint M_{\mathrm{cor};i,j}(\Delta T;t',t'')dt'dt''}{\iint M_{\mathrm{uncor};i,j}(t',t'')dt'dt''} + 1$$

$$= \frac{\iint \tilde{M}_{\mathrm{cor}}(\Delta T;\tau_i,\tau_j)\exp(-t'/\tau_i)\exp(-t''/\tau_j)dt'dt''}{\iint \tilde{M}_{\mathrm{uncor}}(\tau_i,\tau_j)\exp(-t'/\tau_i)\exp(-t''/\tau_j)dt'dt''} + 1$$

$$= \frac{\tilde{M}_{\mathrm{cor}}(\Delta T;\tau_i,\tau_j)}{\tilde{M}_{\mathrm{uncor}}(\tau_i,\tau_j)} + 1 \quad . \qquad (2)$$

When $j = i$, the resulting correlation function $G_{\mathrm{I};i,i}(\Delta T)$ is the autocorrelation of the ith component, and when $j \neq i$, the correlation function $G_{\mathrm{I};i,j}(\Delta T)$ corresponds to the cross-correlation between the ith and jth component. The numerator (the correlated part) and the denominator (the uncorrelated part) can be obtained from the intensity of corresponding peaks in $\tilde{M}_{\mathrm{cor}}(\Delta T;\tau_i,\tau_j)$ and $\tilde{M}_{\mathrm{uncor}}(\tau_i,\tau_j)$, respectively. Note that each fluorescence lifetime component is defined as a component that exhibits a single exponential fluorescence decay with a distinct lifetime, in this description about the principle.

2. Actual extraction of substate-specific correlation functions

2.1. Correlated part

2.1.1. Autocorrelations of N and U, and the cross-correlation between N and I_1.

The correlated parts for the autocorrelations of N and U as well as that for the cross-correlation between N and I_1 can be straightforwardly determined based on equation (2), because the corresponding peaks in $\tilde{M}_{\mathrm{cor}}(\Delta T;\tau',\tau'')$ are well isolated from other peaks. Since a peak appears with a finite width in actual 2D lifetime correlation maps, the area intensity of the peak in $\tilde{M}_{\mathrm{cor}}(\Delta T;\tau',\tau'')$ shown in Fig. 3d-f and Supplementary Fig. 4 were calculated, and the substate-specific autocorrelations of N and U, and cross-correlation between N and I_1 were obtained.

2.1.2. Autocorrelation of I_1

The diagonal peak corresponding to $\mathbf{I_1}$ is overlapped with that of I_2 in I_{en}. Therefore, it is

not possible to extract the peak intensity of I_1 directly from the 2D lifetime correlation map. However, the contributions of I_1 and I_2 in the overlapped peak can be evaluated using independent lifetime distributions ($\alpha(\Delta T;\tau)$) shown in Fig. 3g-i and Supplementary Fig. 5, as described below.

The relationship between $\tilde{M}_{\mathrm{cor}}(\Delta T;\tau',\tau'')$ and $\alpha(\Delta T;\tau)$ is written by equation (3);

$$\tilde{M}_{\mathrm{cor}}(\Delta T;\tau',\tau'') = \sum_{k=1}^{K} \alpha_k(\Delta T;\tau')\alpha_k(\Delta T;\tau''), \qquad (3)$$

where K is the number of independent lifetime distributions at each ΔT. The independent lifetime distribution containing I_1 is easily distinguishable from that including I_2, because I_2 is always associated with I_3 to form a single independent lifetime distribution I_{en} (i.e., I_2 and I_3 are already in equilibrium at the shortest ΔT). Furthermore, I_1 and I_2 always appear in different independent lifetime distribution because no equilibration process between I_1 and I_{en} is observed through whole ΔT. Therefore, the intensity of the diagonal peak of I_1 (i.e., the correlated part for calculating the autocorrelation of I_1) can be evaluated from corresponding independent lifetime distribution (i.e., sp2 in the data for ΔT = 0.2 - 4 μs) by the following way;

$$\tilde{M}_{\mathrm{cor}}(\Delta T;\tau_{\mathrm{I}_1},\tau_{\mathrm{I}_1}) = \sum_{k=1}^{K'} \alpha_{k;\mathrm{I}_1}(\Delta T;\tau_{\mathrm{I}_1})\alpha_{k;\mathrm{I}_1}(\Delta T;\tau_{\mathrm{I}_1}), \qquad (4)$$

where $\alpha_{k;\mathrm{I}1}(\Delta T;\tau)$ and K' correspond to the independent lifetime distribution and the number of independent lifetime distributions that I_1 is contained, respectively. K' needs to be set at 2 for the data at ΔT = 2 - 6, 4 - 8, 6 - 10 and 8 - 12 μs when the equilibration process with N is in progress because I_1 appears in the two independent lifetime distributions. For other ΔTs, K' was set at 1.

2.1.3. Autocorrelation of I_{en}

The correlated parts of the autocorrelation of I_3 and the cross-correlation between I_2 and I_3 can be extracted directly from the relevant isolated peaks in 2D lifetime correlation maps, whereas the correlated part of the autocorrelation of I_2 is obtainable in a similar manner as I_1. However, I_2 and I_3 are in equilibrium even for the shortest ΔT, and I_2 and I_3 behave identically as one single substate I_{en}. Therefore, we evaluated the correlated part of the autocorrelation of I_{en} as a substate that intrinsically exhibits two distinct lifetime components as follows.

Suppose the substate S consists of two lifetime components (τ_1 and τ_2) that are already in equilibrium. Then, the substate-specific autocorrelation function ($G_{I;S,S}(\Delta T)$) can be written as;

$$G_{\mathrm{I;S,S}}(\Delta T) = \frac{\iint M_{\mathrm{cor;S,S}}(\Delta T; t', t'')dt'dt''}{\iint M_{\mathrm{uncor;S,S}}(t', t'')dt'dt''} + 1$$

$$= \frac{\sum_{i=1}^{2}\sum_{j=1}^{2}\iint \tilde{M}_{\mathrm{cor}}(\Delta T; \tau_i, \tau_j)\exp(-t'/\tau_i)\exp(-t''/\tau_j)dt'dt''}{\sum_{i=1}^{2}\sum_{j=1}^{2}\iint \tilde{M}_{\mathrm{uncor}}(\tau_i, \tau_j)\exp(-t'/\tau_i)\exp(-t''/\tau_j)dt'dt''} + 1$$

$$= \frac{\tilde{M}_{\mathrm{cor}}(\Delta T; \tau_1, \tau_1)\tau_1^{\,2} + 2\times\tilde{M}_{\mathrm{cor}}(\Delta T; \tau_1, \tau_2)\tau_1\tau_2 + \tilde{M}_{\mathrm{cor}}(\Delta T; \tau_2, \tau_2)\tau_2^{\,2}}{\tilde{M}_{\mathrm{uncor}}(\tau_1, \tau_1)\tau_1^{\,2} + 2\times\tilde{M}_{\mathrm{uncor}}(\tau_1, \tau_2)\tau_1\tau_2 + \tilde{M}_{\mathrm{uncor}}(\tau_2, \tau_2)\tau_2^{\,2}} + 1. \quad (5)$$

Note that $\tilde{M}(\Delta T; \tau_1, \tau_2)$ and $\tilde{M}(\Delta T; \tau_2, \tau_1)$ are identical. Because the two lifetime components in the substate S are already in equilibrium even for the shortest ΔT, their autocorrelation functions as well as the cross-correlation are the same, that is,

$$\frac{\tilde{M}_{\mathrm{cor}}(\Delta T; \tau_1, \tau_1)}{\tilde{M}_{\mathrm{uncor}}(\tau_1, \tau_1)} = \frac{\tilde{M}_{\mathrm{cor}}(\Delta T; \tau_1, \tau_2)}{\tilde{M}_{\mathrm{uncor}}(\tau_1, \tau_2)} = \frac{\tilde{M}_{\mathrm{cor}}(\Delta T; \tau_2, \tau_2)}{\tilde{M}_{\mathrm{uncor}}(\tau_2, \tau_2)}. \quad (6)$$

Therefore, $G_{\mathrm{I;S,S}}(\Delta T)$ can be rewritten as,

$$G_{\mathrm{I;S,S}}(\Delta T) = \frac{\tilde{M}_{\mathrm{cor}}(\Delta T;\tau_1,\tau_1)}{\tilde{M}_{\mathrm{uncor}}(\tau_1,\tau_1)} + 1 = \frac{\tilde{M}_{\mathrm{cor}}(\Delta T;\tau_1,\tau_2)}{\tilde{M}_{\mathrm{uncor}}(\tau_1,\tau_2)} + 1 = \frac{\tilde{M}_{\mathrm{cor}}(\Delta T;\tau_2,\tau_2)}{\tilde{M}_{\mathrm{uncor}}(\tau_2,\tau_2)} + 1$$

$$= \frac{\tilde{M}_{\mathrm{cor}}(\Delta T;\tau_1,\tau_1) + 2\times\tilde{M}_{\mathrm{cor}}(\Delta T;\tau_1,\tau_2) + \tilde{M}_{\mathrm{cor}}(\Delta T;\tau_2,\tau_2)}{\tilde{M}_{\mathrm{uncor}}(\tau_1,\tau_1) + 2\times\tilde{M}_{\mathrm{uncor}}(\tau_1,\tau_2) + \tilde{M}_{\mathrm{uncor}}(\tau_2,\tau_2)} + 1 \qquad (7)$$

Numerator of the last expression in equation (7) can also be written using independent lifetime distribution of substate S ($\alpha_{\mathrm{S}}(\Delta T;\tau)$);

$$G_{\mathrm{I;S,S}}(\Delta T) = \frac{\left(\alpha_{\mathrm{S}}(\Delta T;\tau_1) + \alpha_{\mathrm{S}}(\Delta T;\tau_2)\right)^2}{\tilde{M}_{\mathrm{uncor}}(\tau_1,\tau_1) + 2\times\tilde{M}_{\mathrm{uncor}}(\tau_1,\tau_2) + \tilde{M}_{\mathrm{uncor}}(\tau_2,\tau_2)} + 1 . \qquad (8)$$

Therefore, the correlated part for the autocorrelation of substate S can be evaluated using corresponding independent lifetime distribution. Using equation (8), the correlated part of the autocorrelation of $\mathrm{I_{en}}$ was evaluated from the corresponding independent lifetime distribution.

3. Uncorrelated part

The uncorrelated part (i.e., the denominator of equation (2) and (8)) can be evaluated from the amplitude of each substate in the ensemble-averaged fluorescence decay that was calculated from all photon data because they have the following relationship (see also equations (4)-(7) in Methods);

$$\tilde{M}_{\mathrm{uncor}}(\tau_i,\tau_j) = A(\tau_i)\frac{T_0 - \Delta T}{T_0}\cdot\frac{\Delta\Delta T}{T_0}A(\tau_j) , \qquad (9)$$

where $A(\tau_i)$ is the amplitude of τ_i component in the ensemble-averaged fluorescence decay, T_0 is the total measurement time, ΔT is macrotime delay and $\Delta\Delta T$ is the width of temporal window. In case of $\mathrm{I_{en}}$, denominator in equation (8) can be written as;

$$\tilde{M}_{\mathrm{uncor}}(\tau_{\mathrm{I2}},\tau_{\mathrm{I2}}) + 2\times\tilde{M}_{\mathrm{uncor}}(\tau_{\mathrm{I2}},\tau_{\mathrm{I3}}) + \tilde{M}_{\mathrm{uncor}}(\tau_{\mathrm{I3}},\tau_{\mathrm{I3}})$$

$$= \frac{T_0 - \Delta T}{T_0} \cdot \frac{\Delta\Delta T}{T_0} \left(A(\tau_{I2})^2 + 2A(\tau_{I2})A(\tau_{I3}) + A(\tau_{I3})^2 \right)$$

$$= A_{\text{Ien}}^{2} \frac{T_0 - \Delta T}{T_0} \cdot \frac{\Delta\Delta T}{T_0} . \qquad (10)$$

In equation (10), A_{Ien} corresponds to the amplitude of substate I_{en} in the ensemble-averaged fluorescence decay. Therefore, the uncorrelated part of each substate component can be evaluated by calculating the corresponding amplitude in the ensemble-averaged fluorescence decay.

To evaluate the amplitude of each substate in the ensemble-averaged fluorescence decay, the fluorescence decay curve of each substate was first calculated by Laplace transform of the corresponding independent lifetime distributions (sp1, sp2, sp3 and sp4 in Fig. 3g, which correspond to N, I_1, I_{en} and U, respectively). After the amplitude at the time origin is normalized to 1, the normalized decay curves were used to fit the ensemble-averaged fluorescence decay curve for obtaining the absolute amplitude of each substate. In the fitting, the ratio between N and I_1 was kept to 0.25 which was determined from the intensity ratio of the diagonal peaks of N and I_1 in $\tilde{M}_{\text{cor}}(0.2 - 4\mu s; \tau', \tau'')$. The obtained amplitudes were used for calculating the uncorrelated parts using equation (9) or (10).

Supplementary Note 5. Origin of the dark states and validity of the Scheme involving them:

The highly quenched state, which is also called the dark state, has already been reported for some organic dyes attached to proteins[3,4]. The quenching mechanism has also been studied, and it was suggested that the ground-state complex is formed and that photo-induced electron transfer occurs between the dye and aromatic residues such as tryptophan or tyrosine. In fact, cyt *c* has one tryptophan and five tyrosines in its sequence. Therefore, it seems possible that cyt *c* takes conformations in which specific interaction between Alexa546 and these aromatic residues is significant and it quenches fluorescence very efficiently. The fluorescence lifetimes of such highly quenched states have been reported to be several ps[4], which is too short to be detected with the TCSPC system employed in the present study.

We modify simple Scheme 1 (Fig. 4) and propose Scheme 2 (Fig. 6) on the basis of the correlation functions of each substate derived from 2D lifetime correlation maps at pH 3.5. We also have experimental grounds for Scheme 2, especially those about the involvement of dark states, in the data taken at different pH.

Supplementary Figure 6a depicts the fluorescence intensity correlation and the lifetime-weighted fluorescence correlation of Alexa546_cyt *c* at pH 1.0 where the contribution of the unfolded state (U) is predominant. The correlation curves of Alexa546_DTT in the same pH condition are also shown for comparison. The fluorescence intensity correlation and lifetime-weighted fluorescence correlation become identical when the sample only contains a single species (from a viewpoint of the fluorescence lifetime)[5], as they are exactly the same for Alexa546_DTT. Also for Alexa546_cyt *c*, the two correlation curves are very close, reflecting that the contribution of the unfolded state (U) is predominant and that the system is quite

homogeneous at pH 1.0. The small difference recognized between two correlation curves is due to minor contributions from intermediate states as shown in Supplementary Fig. 2b. However, the two correlation curves of Alexa546_cyt *c* exhibit a steep slope in the short ΔT region, which is completely missing in the correlation curves of Alexa546_DTT. This marked difference clearly shows that the correlation curves of Alexa546_cyt *c* at this pH cannot be described only by translational diffusion. Because the system is quite homogeneous from a view point of the fluorescence lifetime but there is a feature indicating chemical reactions, the reaction should be a reaction between the fluorescent state and the dark state.

In Supplementary Figure 6b, pH dependence of the fluorescence decay of Alexa546_cyt *c* is shown. For this data set, the experimental conditions such as the protein concentration, excitation intensity, and data accumulation time are all the same. Therefore, we can directly compare their time-resolved fluorescence intensities. Remarkably, the intensity at $t = 0$ decreases drastically by changing pH from 5.5 to 3.5, and then increases by further shifting pH to 2.5. The fluorescence intensity at $t = 0$ directly reflects the total population of the emissive Alexa546 that is detectable with the TCSPC system. Thus, if the intensity at $t = 0$ changes, it means that the total population of emissive Alexa546 changes with change of pH. Because the total population of Alexa546 is the same, it implies the involvement of highly quenched states in the folding scheme. This also supports Scheme 2 shown in Fig. 6.

Supplementary References

1 Ishii, K. & Tahara, T. Two-dimensional fluorescence lifetime correlation spectroscopy. 1. Principle. *J. Phys. Chem. B* **117**, 11414-11422 (2013).

2 Ishii, K. & Tahara, T. Two-dimensional fluorescence lifetime correlation spectroscopy. 2. Application. *J. Phys. Chem. B* **117**, 11423-11432 (2013).

3 Marme, N., Knemeyer, J. P., Sauer, M. & Wolfrum, J. Inter- and intramolecular fluorescence quenching of organic dyes by tryptophan. *Bioconjugate Chem.* **14**, 1133-1139 (2003).

4 Sun, Q. F., Lu, R. & Yu, A. C. Structural heterogeneity in the collision complex between organic dyes and tryptophan in aqueous solution. *J. Phys. Chem. B* **116**, 660-666 (2012).

5 Ishii, K. & Tahara, T. Resolving inhomogeneity using lifetime-weighted fluorescence correlation spectroscopy. *J. Phys. Chem. B* **114**, 12383-12391 (2010).
